# Stress granules regulate paraspeckles: RNP granule continuum at work

**DOI:** 10.15698/cst2019.12.207

**Published:** 2019-11-21

**Authors:** Haiyan An, Tatyana A. Shelkovnikova

**Affiliations:** 1 Medicines Discovery Institute, Cardiff University, Cardiff, CF10 3AT, United Kingdom.

**Keywords:** RNP granule, stress granule, paraspeckle, nuclear body, ALS

## Abstract

Eukaryotic cells contain several types of RNA-protein membraneless macro-complexes – ribonucleoprotein (RNP) granules that form by liquid-liquid phase separation. These structures represent biochemical microreactors for a variety of cellular processes and also act as highly accurate sensors of changes in the cellular environment. RNP granules share multiple protein components, however, the connection between spatially separated granules remains surprisingly understudied. Paraspeckles are constitutive nuclear RNP granules whose numbers significantly increase in stressed cells. Our recent work using affinity-purified paraspeckles revealed that another type of RNP granule, cytoplasmic stress granule (SG), acts as an important regulator of stress-induced paraspeckle assembly. Our study demonstrates that despite their residency in different cellular compartments, the two RNP granules are closely connected. This study suggests that nuclear and cytoplasmic RNP granules are integral parts of the intracellular “RNP granule continuum” and that rapid exchange of protein components within this continuum is important for the temporal control of cellular stress responses. It also suggests that cells can tolerate and efficiently handle a certain level of phase separation, which is reflected in the existence of “bursts”, or “waves”, of RNP granule formation. Our study triggers a number of important questions related to the mechanisms controlling the flow of RNP granule components within the continuum and to the possibility of targeting these mechanisms in human disease.

Eukaryotic cells contain a variety of large, multicomponent RNA-protein complexes termed ribonucleoprotein (RNP) granules. RNP granules represent membraneless organelles – biomolecular condensates lacking a membrane and maintained through an intricate network of protein-protein, protein-RNA and RNA-RNA interactions. RNA-binding proteins with prion-like domains in their structure drive liquid-liquid phase separation and formation of a functional cellular compartment. High local concentration of molecules within RNP granules increases the efficiency of biochemical processes and thus makes these structures ideal platforms for the regulation of numerous processes involving RNA. Because of their remarkable molecular plasticity, RNP granules are also widely utilised by cells to rapidly adjust and rewire regulatory networks in response to stress, serving as both stress sensors and effectors. The best characterised stress-induced RNP granules are stress granules (SGs) - large, amorphous cytoplasmic entities induced by various stresses *in vitro* and *in vivo*. RNP granules are also present in the nucleus, and nuclear RNP granules are often called “nuclear bodies”. The paraspeckle is a prototypical nuclear body found in the majority of cultured cells and in some cell types *in vivo*, whose exact biological functions remain poorly understood. Paraspeckle numbers are low in naïve cells but can increase many-fold in stressed cells.

Due to their distinct localisation, nuclear bodies and cytoplasmic RNP granules have long been considered independent entities. Indeed, paraspeckles and other nuclear bodies do not form outside the nucleus, whereas SGs are exclusively cytoplasmic. However, our recent study has revealed that paraspeckles and SGs are structurally and functionally connected. In particular, SGs have been found to act as important regulators of stress-induced paraspeckle assembly in response to diverse stress signals. Our study has two important implications. Firstly, it suggests that different types of RNP granules are parts of a continuum of interconnected membraneless organelles. Redistributing components of RNP granules to the most appropriate cellular locations during stress would eliminate the need in their degradation and re-synthesis. The flow of protein (and possibly, RNA) components between RNP granules would therefore constitute an efficient mechanism of post-translational control of cellular responses to diverse changes in the environmental conditions.

Secondly, our study has demonstrated that cells employ “bursts”, or “waves”, of phase-separation coupled to RNP granule formation during stress. At least two waves of RNP granule formation occur in response to acute stress – one in the cytoplasm (SGs) followed by the second one in the nucleus (paraspeckles) **(Fig. 1A)**. Such a biphasic mode of RNP granule assembly is seemingly crucial for differential adjustment of the cellular metabolism during the acute and recovery phases of stress. It is possible that further waves of RNP granule assembly exist, e.g. other nuclear bodies may hyper-assemble late during the recovery **(Fig. 1A)**. While the above model is applicable to a wide range of acute stresses, it is not clear whether it will likewise work in chronic stresses characterised by SG and paraspeckle assembly, for example, in viral infection. Indeed, in cells infected by some types of viruses, stress response and SG assembly oscillate which allows building up efficient antiviral response whilst keeping the cell alive. It would be important to establish whether SGs and paraspeckle responses manifest as antiphase oscillations during chronic stresses **(Fig. 1B)**.

**Figure 1:**
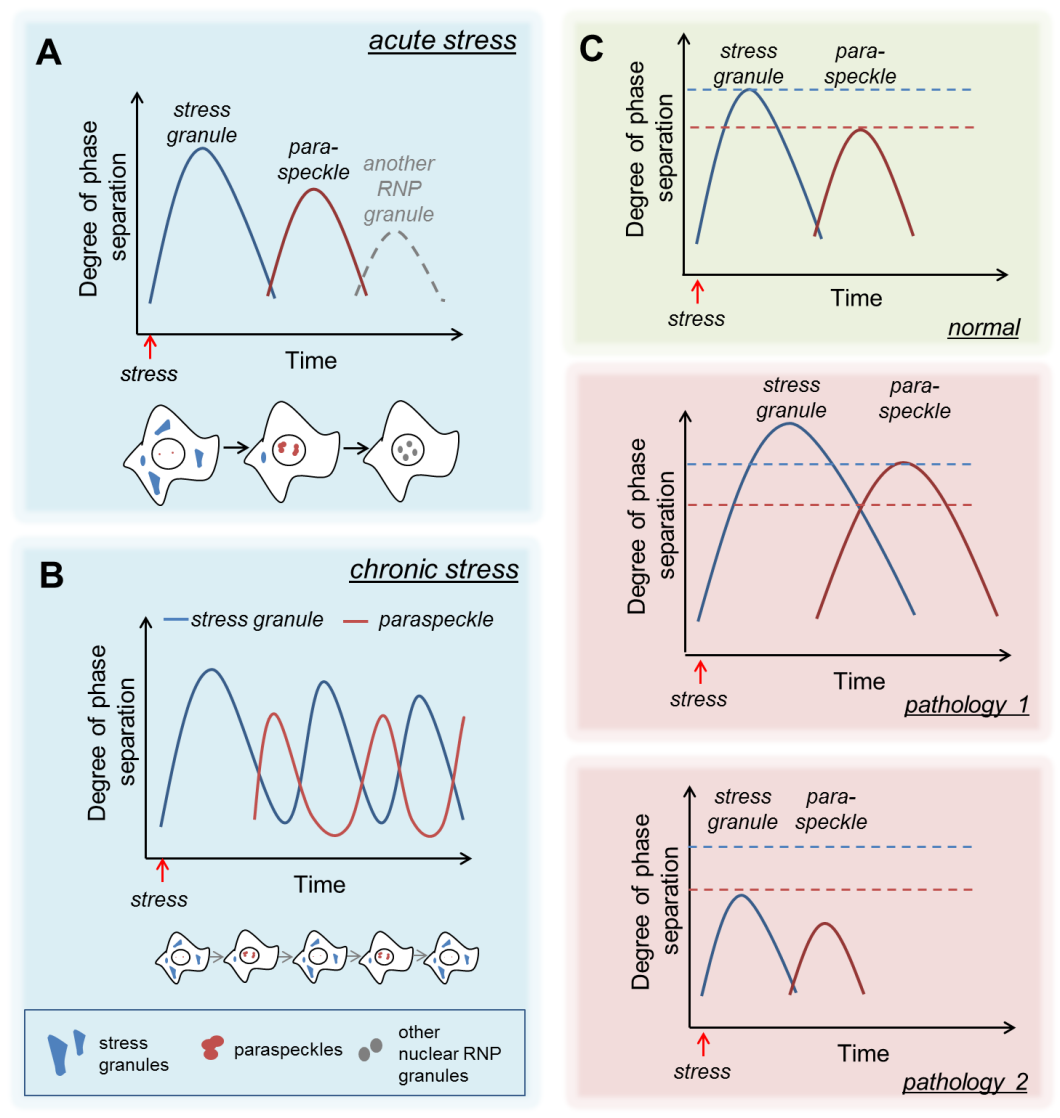
FIGURE 1: “Waves” of phase separation/RNP granule formation in response to acute (A) and chronic (B) stress as well as in disease states (C). See text for details.

Because of the high local concentration of biomolecules inside RNP granules, weakened or enhanced interactions between their components can lead to severe dysfunction of RNP granules and disease states. For example, SGs have been linked to amyotrophic lateral sclerosis/frontotemporal dementia (ALS/FTD) pathology and have been found to serve as drivers of oncogenic growth. Likewise, paraspeckle dysfunction is implicated in multiple forms of cancer and more recently, has been linked to ALS/FTD. Our study raises an intriguing possibility that during stress, a cell can only handle a certain “degree” of phase separation (initially in the cytoplasm and subsequently in the nucleus but not simultaneously) during stress. In this scenario, exceeding this phase separation “threshold” – due to a mutation in an aggregate-prone RNA-binding protein, malfunction of signalling molecules and/or RNP granule disassembly factors – would cause abnormal stabilisation of RNP granules, with their subsequent persistence and associated toxicity, as seen in ALS/FTD **(Fig. 1C**, pathology 1). An opposite situation is also possible, when RNP granule assembly is attenuated (e.g. by a mutation disrupting phase separation) and the requisite degree of phase separation is not achieved **(Fig. 1C**, pathology 2). In the latter case, impaired SG assembly would be coupled with reduced paraspeckle formation at the later stages of stress/during recovery which may result in compromised stress signalling.

SGs and paraspeckles both represent excellent model RNP granules suitable for the analysis of molecular underpinnings of RNP granule regulation under physiological and pathological conditions. To improve our understanding of the RNP granule continuum, a number of questions will have to be addressed: i) What constitutes the SG-regulated paraspeckle regulome and how is it controlled under stress? ii) Are other RNP granules regulated by SGs in a similar fashion? iii) Is this regulation stress-specific? iv) Does this regulation contribute to cell fate decisions? v) Which of the disease subtypes are characterised by reduced and which - by enhanced RNP granule cross-regulation? Ultimately, this knowledge can be harnessed to restore the altered RNP granule homeostasis in human disease and guide therapeutic developments for age-related neurological diseases and cancer.

